# 72 Craniotomy Surgical Site Infection Cluster: Identification, Response, and Prevention Measures

**DOI:** 10.1017/ash.2026.10502

**Published:** 2026-06-23

**Authors:** Shanmukha Priya Peddireddy, Valerie Cluzet, Ali Al Safi, Fadila Noor, Rossana Seno, Hector Ojeda-Martinez, Kelcey Noble, Akash Gupte, Zayd Anwar, Wasif Shah

**Affiliations:** 1 Northwell health; 2 Northwell Health/Nuvance Health; 3 The Ohio State University Wexner Medical Center; 4 University of Rochester Medical Center; 5 Bridgeport Hospital; 6 NYU Langone

## Abstract

**Background and Objective:** Antipseudomonal antibiotics are frequently initiated empirically in hospitalized patients with suspected pneumonia. However, de-escalation often remains suboptimal. Behavioral “nudge” interventions in microbiology reporting have shown potential to enhance antimicrobial stewardship. In June 2022, our institution implemented a revised microbiology comment in sputum culture result reporting emphasizing “commensal respiratory flora only: no MRSA or P. aeruginosa”. This study aimed to evaluate whether this reporting nudge increased de-escalation of antipseudomonal therapy within 24 hours of final respiratory culture results. **Methods:** We conducted a retrospective cohort study of adults (<18 years) admitted between December 1, 2021 - May 31, 2022 (pre-intervention) and July 1–December 31, 2022 (post-intervention) who had respiratory cultures obtained and were started on at least one antipseudomonal antibiotic within 48 hours of the culture being sent. Patients were excluded if cultures grew P. aeruginosa, if another infection required antipseudomonal coverage, if therapy was required due to allergies, or if they had neutropenic fever. The primary outcome was de-escalation, defined as discontinuation or narrowing of antipseudomonal therapy, within 24 hours of the finalized respiratory culture report. Secondary outcomes included duration of therapy, hospital length of stay, Clostridioides difficile infection within 30 days, and in-hospital mortality. Outcomes were compared in pre- and post-intervention cohorts. **Results:** Of 230 screened patients, 175 met the inclusion criteria (107 pre-intervention, 68 post-intervention). Approximately one-third of subjects had community-acquired pneumonia(34 in pre-intervention and 28 in post-intervention). Baseline characteristics suggested the pre-intervention cohort had higher severity of illness, with more ICU admissions (N =68 in pre-intervention and N=28 in post intervention) and more hospital-acquired (N= 21in pre and N=8 in post) or ventilator-associated pneumonia (N=7 in pre, N=2 in post). De-escalation within 24 hours occurred in (76) 71% of pre-intervention patients and (50) 73.5% post-intervention (p=0.72). Median duration of antibiotics after final culture result was 1 day (IQR, -1,3) in the pre-intervention group and 0 days (IQR, -2,3) in the post-intervention group (P=0.36). Median hospital length of stay was significantly longer in the pre-intervention group (IQR 13(6,20) vs 9 (5,16); p=0.03). Rates of C. difficile infection (<5%) and in-hospital mortality did not differ significantly between groups. **Conclusion:** Implementation of a microbiology nudge comment in respiratory culture result reporting did not significantly increase antipseudomonal antibiotic de-escalation. High rates of de-escalation were observed even prior to the intervention. Explanations for the finding may be that respiratory cultures are being sent unnecessarily and, therefore, they are not being used as data to act upon and/or anti-pseudomonal antibiotics are being started empirically unnecessarily, particularly in patients with CAP. The cohorts did differ in severity of illness, which may have impacted the results. These findings highlight the need for additional education regarding guideline-directed respiratory culture utilization and empiric antibiotic choices.

